# Orthostatic-dependent cardiopulmonary responses among trained females during intensity matched resistance exercises in a pilot randomized crossover study

**DOI:** 10.1038/s41598-025-04383-9

**Published:** 2025-06-23

**Authors:** Johannes Lässing, Stefan Hochstein, Maren Witt, Roberto Falz

**Affiliations:** 1 Department of Exercise Science & Sports Medicine, Martin Luther University Halle-Wittenberg, von-Seckendorff-Platz 2, D-06120 Halle (Saale), Germany; 2 Human‒Machine-Interaction, Magdeburg-Stendal University of Applied Science, Breitscheidstraße 2, 39114 Magdeburg, Germany; 3 Department of Sports Biomechanics, University of Leipzig, Jahnallee 59, D-04109 Leipzig, Germany; 4 Department of Movement Science, Martin Luther University Halle-Wittenberg, von-Seckendorff-Platz 2, D-06120 Halle (Saale), Germany; 5 Institute of Sports Medicine & Prevention, University Leipzig, Rosa- Luxemburg-Straße 30, 04109 Leipzig, Germany

**Keywords:** Intensity-matched exercise, Cardiac and vascular exercise regulation, Resistance training, Preventive medicine, Hypertension

## Abstract

The impact of orthostatic regulation during exercise, particularly resistance training, is not fully understood. This study investigates the acute cardiopulmonary responses of intensity-matched resistance exercises, targeting similar muscle groups but performed in different body positions in young trained females. Fourteen healthy females (21.6 ± 2.0 years) performed a 3-repetition Maximum test (3-RM) for the squat movement in the Smith machine (SM) and the leg press (LP). During two subsequent visits, they randomly completed two training sessions in SM and LP (two sets of ten repetitions at 50% 3-RM). Blood pressure (vascular unloading technique) and cardiopulmonary parameters (impedance cardiography, spirometry) were measured continuously. At baseline, there was a significant difference in heart rate and stroke volume between the SM and LP conditions. During training sessions, the SM condition showed higher ground reaction force (986.9 ± 93.3 vs. 811.2 ± 71.6 N; *p* < .01), systolic blood pressure (156 ± 15 vs. 141 ± 10 mmHg; *p* < .01), diastolic blood pressure (111 ± 11 vs. 96 ± 8 mmHg; *p* < .01), HR (123 ± 11 vs. 97 ± 7 bpm; *p* < .01), and oxygen uptake (901 ± 104 vs. 623 ± 65 ml/min; *p* < .01) compared to the LP condition. Total peripheral resistance (TPR) was similar. Significant different post-exercise changes could be detected in mean arterial pressure (-20.9 ± 9.9 vs. 3.3 ± 11.0 mmHg; *p* < .01) and TPR (-2.3 ± 1.7 vs. 0.7 ± 1.7 mmHg⋅ l⋅min-1; *p* < .01). Squats in the SM require greater cardiovascular and pulmonary effort than matched exercising in LP due to orthostatic stress and higher muscle activation. Conversely, the risk of blood pressure peaks is much lower with LP. Future analysis should focus on the effects of body position on patient responses.

## Introduction

The evidence consistently demonstrates the effectiveness of strength training programs in enhancing muscle strength, strength endurance, and muscle mass, as well as in reducing the cardiac risk profile^1^. The American Heart Association and the Association of Scientific Medical Societies in Germany recommend that strength training for cardiac rehabilitation should involve more repetitions at an intensity of 40–60% of the one-repetition maximum (1RM). Additionally, maintaining the ischemic safety threshold specified by the rate pressure product (RPP) is recommended, which should be below 36,000 ^1–4^. Compared with endurance training, resistance training has smaller effects on cardiopulmonary parameters, including peripheral systolic (SBP) and diastolic (DBP) blood pressure^5–7^. On the other hand, intense, isolated strength training has been shown to induce high-pressure responses. These factors may lead to chronic morphological adaptations, significantly increasing relative ventricular wall thickness while maintaining a constant ventricular internal diameter (LVD)^8–11^. Meta-analyses by Cornelissen et al. (2005) and Edwards et al. (2023) indicate that isotonic strength training is less effective in reducing resting blood pressure than isometric training is^12,13^. Nevertheless, attention should be focused on high blood pressure peaks in certain groups, which occur particularly during high exertion during the Valsalva manoeuvre^14^.

Squats and leg press (LP) exercises are commonly performed during strength training. While both exercises activate similar muscle groups, standing squats generate significantly greater muscle activity in the knee and hip extensors, which enhances jumping power and leads to greater muscle growth than LP exercises do^15–19^. A systematic review by Blazek et al. (2019) revealed that peak intra-abdominal pressure was significantly greater during a stand-squat exercise performed at an intensity exceeding 90% of the one-repetition maximum (219 ± 19.5 mmHg with a force of 1590–1764 N) than during a LP exercise performed at 100% of the one-repetition maximum for four repetitions (161 ± 55 mmHg with a force of 1520 ± 282 N). The authors concluded that body position (seated or standing) affects the pressure response. The LP poses a lower risk than squats do, as it elicits a significantly reduced intra-abdominal pressure response^20^. The effect of body position on heart rate (HR) and blood pressure at rest is well documented, with the baroreflex playing a crucial role in maintaining arterial pressure^21–23^. However, the influence of body position on the cardiopulmonary response during exercise remains poorly understood. To our knowledge, no prior study has directly compared intensity-matched LP exercises with squats, which could provide valuable insights into differences in cardiopulmonary modulation during dynamic exercise. The primary objective of our study was to investigate whether body position affects the acute cardiopulmonary response to intensity-matched squat tasks performed with a Smith machine (SM) or a LP machine among healthy young females. Achieving comparable muscle intensity between the two types of exercise is challenging^18^.Thus, the exercises are tailored to each individual’s three-repetition maximum (3-RM), as the American Heart Association recommended for cardiac rehabilitation^1,2^. In this initial examination with healthy female participants, we aimed primarily to identify potential differences in cardiopulmonary responses to various body positions during strength exercises. To address known differences in cardiovascular regulation between genders and minimize potential sex-specific variations in cardiopulmonary function, this study focuses specifically on a cohort^24,25^. This is important since women are rarely studied separately.

Based on the differences in position-dependent regulation during resting conditions and the findings of Blazek et al. (2019), we hypothesized that the cardiac and blood pressure responses would vary between intensity-matched standing squats in the Smith Machine (SM) and seated leg press (LP) training (at 50% of 3-RM) during and after exercise^20,26^. Assessing body position may help assess the risks and potential adaptations of specific resistance exercises and their derivatives for cardiac rehabilitation.

## Methods

### Participants

This within-subject, randomized crossover pilot study was conducted in accordance with the latest version of the Declaration of Helsinki and received approval from the Ethics Committee of the Medical Faculty at the University of Halle (Saale) (2023–202) between November 2023 and April 2024. Given the exploratory nature of the study, our sample size was determined according to the specifications for a pilot study^27^. Written informed consent was obtained from all participants before their enrolment. The study group comprised 14 homogeneous, healthy and trained females (handball players from the Junior Team SV Union Halle-Neustadt) with an average age of 21.6 ± 2.0 years, body mass of 68.9 ± 6.5 kg, height of 170.4 ± 4.8 cm, BMI of 23.6 ± 1.6 kg/m², fat mass of 27.9 ± 4.2%, and muscle mass of 47.0 ± 3.2%. Nine of the fourteen players used hormonal contraception while five did not. The average training volume per week was 9.0 ± 1.0 h. On average, female participants engage in 1.5 h of circuit training each week, using their body weight. The participants lack general experience with the strength machines being used. The exclusion criteria included (1) cardiac, pulmonary, or inflammatory diseases; (2) athletic inactivity; and (3) any orthopedic anomalies identified during the assessments.

### Study design

**Fig. 1:**
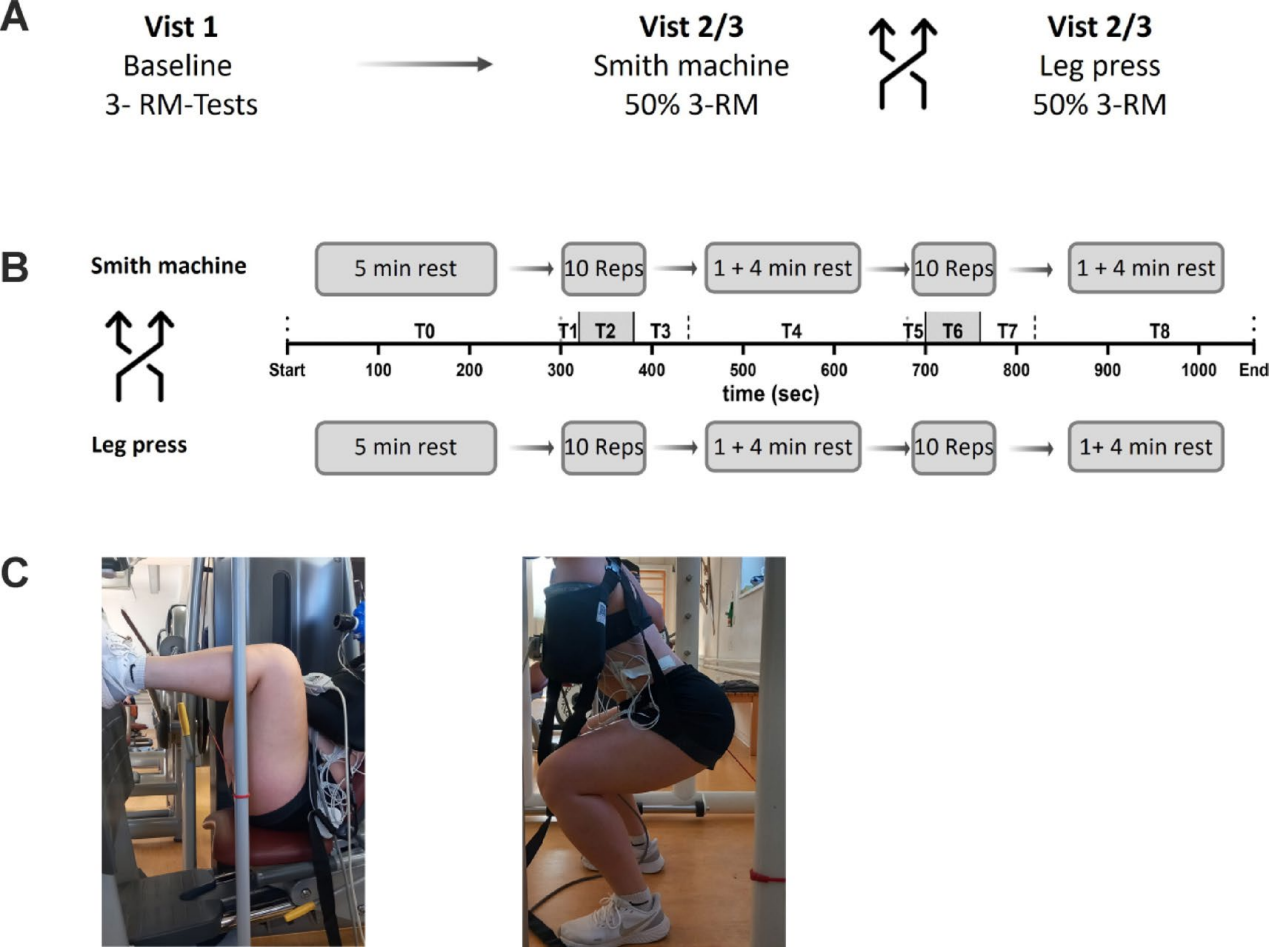
(**A**) Timeline of study visits; (**B**) Timeline of training sessions of squats in the Smith machine and leg bends in the Leg Press. T0, rest period before sets; T1 and T5, isometric period before sets; T2 and T6, set with 10 repetitions; T3 and T7, immediate postexercise period; T4 and T8 postexercise period; (**C**) the individual end position in the Smith machine and leg press shows a 90-degree knee angle.

This study involved three visits (see Fig. 1A). During the first visit, participants underwent a baseline assessment. This included collecting medical history data, administering a lifestyle questionnaire, and assessing physical activity status, tobacco use, and alcohol consumption. Furthermore, body composition was assessed via a bioelectrical impedance scale (Tanita BC-545 N, Tanita Europe B.V., Netherlands). After that, the 3-repetition maximum (3-RM) of each participant was determined via a Smith machine (SM, Technogym Germany GmbH, Germany) and the leg press (LP, Selection 700 Leg Press, Technogym Germany GmbH, Germany) via the approximation method. This method determines the maximum weight that can be lifted for three repetitions with the correct technique^28^.

During visits 2 and 3, the participants randomly performed two strength training sessions using either the SM or the LP (described below). The visits occurred at the same time of day to minimize circadian influences. A minimum two-day rest period was scheduled between visits to ensure adequate recovery. During each training session, the participants performed two sets of 10 repetitions at 50% of their 3-RM intensity. Before each visit, the participants were instructed to avoid any lower-body resistance training for 48 h before the testing days and to adhere to a standardized nutrition plan. During the study visits, none of the participants reported any physical complaints.

### 3-repetition maximum test

The 3-RM tests followed the American College of Sports Medicine (ACSM) guidelines for 1-RM tests^28^. After the 3-RM in the SM was assessed, the participants were allowed to rest for 45 min to minimize the effects of fatigue. Following this rest period, the 3-RM in the LP was determined. Both exercises were performed with maximal knee flexion of 90 degrees. To ensure that the participants achieved a 90-degree angle during the 3-RM test and exercise sessions for the squat, a visual limitation (rubber band) was mounted for orientation (Fig. 1C). The distance from the femoral head in the starting position in the SM (fully extended knee) to the ground was measured. The distance to the ground during the 90-degree squat position was subsequently measured. The difference between these measurements represents the range of motion, which we then applied to the LP condition as well.

The LP was set with the upper body upright at a 60-degree angle. The participants were instructed to place their feet symmetrically and parallelly on the footplate, with a slight external rotation of just under 5 degrees.

The test procedure commenced with a warm-up and familiarization phase, during which the participants performed ten repetitions of the respective exercise without any load, followed by five repetitions with a submaximal load.

The 3-RM (three-repetition maximum) was determined through single trials with incrementally increasing loads. If the participants performed three technically correct repetitions, the load was increased in the next attempt, with four minutes of rest between each attempt. If three clean repetitions were not completed at a certain load level, the previous load was recorded as the 3-RM.

### Strength training sessions

All participants completed a five-minute warm-up on a cycle ergometer set at 50 watts and 75 rpm. This was followed by ten repetitions of the respective resistance exercise (SM or LP) without an external load. During this phase, the participants familiarized themselves with the breathing instructions (inhaling during the eccentric phase and exhaling during the concentric phase) as well as the visual and auditory cues (Interval Timer, dreamspark), which were used in the subsequent exercise sessions.

Before each set, the participants were instructed to hold the weight isometrically in the starting position while the ground reaction force was measured (T1/T5; Fig. 1B). During each training session (SM and LP), two sets of 10 repetitions (T2 and T6; each 60 s; Fig. 1B) were completed with a 5-minute rest period (T3 & T4 and T7 & T8; Fig. 1B) between each set^29^^30^. This ensured cardiopulmonary and metabolic recovery and facilitated the analysis of the postexercise period. The execution of each squat was timed to achieve a target of 2 s for the descent, 2 s for the ascent, and a 2-second hold at the top, in total 6 s per repetition. We also assessed breathing patterns, instructing participants to inhale during the eccentric phase and exhale during the concentric phase^1^. Each participant received visual and auditory feedback on their performance in completing the task correctly. The movement execution and range of motion during training sessions were standardized according to the 3-TM test described above. To ensure the safety of our participants during the one-armed execution in the Smith machine and to facilitate a clean execution of the exercise, we limited the number of sets to two.

### Physiological measurements during strength training sessions (internal effort, visits 2 and 3)

Continuous noninvasive blood pressure measurements (contB) were obtained from the finger via the vascular unloading technique (Task Force Monitor 340i, CNSystems Medizintechnik GmbH, Austria). The mean values were calculated for each exercise session, which consisted of two sets lasting one minute each. The mean immediate postexercise values were taken one minute after each exercise session. Additionally, the mean postexercise values were collected over five minutes following the exercise sessions. Continuous noninvasive arterial pressure monitoring is consistent with intermittent oscillometric measurements^31–34^. Cardiac output (CO), stroke volume (SV), end-diastolic volume (EDV), ejection fraction (EF), and heart rate (HR) were continuously measured via impedance cardiography (PhysioFlow, Manatec BioMedical, France) at a sampling interval of 5 s. Oxygen consumption (VO_2_), end-tidal oxygen partial pressure (PetO_2_), end-tidal carbon dioxide partial pressure (PetCO_2_), respiratory rate (RR), tidal volume (VT), and minute ventilation (V_E_) were measured breath-by-breath using a spirometer (Metalyzer 3B, Cortex Biophysik GmbH, Germany) at 5-second intervals. Ratings of perceived exertion (RPE), on a scale from 1 to 10 (with 10 indicating total exhaustion), were recorded at baseline, at the end of each set, and after three minutes of recovery. All outcomes were obtained at baseline, as well as during and after the training sessions. Six disposable sensors were placed on the neck and chest for impedance cardiography to detect electrical and impedance changes in the thorax caused by cardiac flow. The electrodes were applied following standard procedures after the skin was prepared by peeling and disinfecting it. They were calibrated according to the manufacturer’s instructions before each training session. For beat-to-beat finger measurements, oscillometric blood pressure measurements were performed for calibration before and during each break (after 3 min). We consistently used the left arm for measuring oscillometric blood pressure while the right arm was secured in an arm sling at a 90-degree angle.

For editing purposes, we averaged the values at 5-second intervals. We calculated the mean values for two sets (2 × 60 s; T2 and T6) and the remaining periods (2 × 275 s; T0 and T4, excluding the isometric period). Additionally, we analysed the postexercise period (2 × 275 s; T4 and T8). To compare the immediate postexercise response, we also examined the mean value of the first 60 s (2 × 60 s; T3 and T7) following each set.

The rate pressure product (RPP) was calculated using SBP and HR. Stroke work (SW) was measured in Joules (J) and calculated according to the formula SW = SV × MAP/7.5 (MAP mean arterial pressure)^35^. Total peripheral resistance (TPR) was determined via continuously measured mean arterial blood pressure and CO (TPR = MAP/CO). Work efficiency rate [(physical work J/metabolic effort J) * 100] was calculated according metabolic effort [VO_2_ l/min _work_ * 20,2 kJ/litre O_2_ = J] and physical work for ten repetitions (GRF _work_ N * distance _work_ m * 10 _repetitions_).

### Biomechanical measurements during the 3-RM test and training sessions (external effort, visits 1–3)

The ground reaction force (in Newtons) during the strength training sessions was measured via force-measuring soles (Loadsol, Novel GmbH, Germany). To standardize the force measurements, all the participants wore the same type of shoes (Vty Sneakers, China). The execution sequence was provided as visual and auditory feedback via an interval timer. The theoretical work (in Joules) was calculated on the basis of the static ground reaction force (in Newtons), the recorded distance (in meters), and the number of repetitions completed (10 squats).

### Statistical analysis

Unless otherwise stated, all values are expressed as the means and standard deviations. The significance level was set at alpha < 0.05, and all tests were two-tailed. The data were analysed via Microsoft Office Excel^®^ 2007 for Windows (Microsoft Corporation, Redmond, Washington, USA) and GraphPad Prism 9 for Windows (GraphPad Software Inc., California, USA). The D’Agostino‒Pearson normality test was employed for distribution analysis. If a normal distribution was confirmed, statistical comparisons between sessions were conducted via two-way repeated-measures ANOVA, followed by Bonferroni post hoc correction for multiple comparisons. To assess cardiopulmonary changes, the mean differences between individual periods (∆ = current period - previous period) were compared via two-way ANOVA with repeated measures. Within-group differences for the 3-RM tests were assessed with a paired Student’s t test. When the data were not normally distributed, the nonparametric Wilcoxon matched-pairs signed rank test was used. Changes from rest to load as a function of ground reaction force (∆outcome/GRF) were also analysed via paired Student’s t tests.

## Results

### Ground reaction force

Table 1 presents the results of the 3-RM test. The mass of the external load applied (in kg) is greater for the LP condition than for the SM condition. The 3-RM results indicated that the ground reaction force was significantly greater during the LP than during the SM. Furthermore, when performing at 50% of the 3-RM, the force values and work performance measured in joules (J) were significantly greater in the SM than in the LP. Additionally,

**Table 1:** Mean isometric force and ground reaction force during SM and LP (*n* = 14; mean).

3-RM test	SM	LP	*p* value
External load (weight disks. kg)	**75.6 ± 2.9**	**106.4 ± 10.6**	*p* <.01
Ground reaction force (incl. body weight, N)	**1261 ± 129.9**	**1411 ± 116.6**	*p* <.01
Borg scale (1–10)	9.8 ± 0.3	9.9 ± 0.3	p.61
**50% 3-RM test**			
External load (weight disks, kg)	**37.8 ± 9.0**	**53.9 ± 7.1**	*p* <.01
Ground reaction force (incl. body weight, N)	**986.9 ± 93.3**	**811.2 ± 71.6**	*p* <.01
Work efficiency rate (%)	**12.6 ± 2.4**	**14.7 ± 2.9**	*p* <.01
Rate Pressure product (HR x RRsys)	**28,354 ± 3392**	**20,390 ± 2338**	*p* <.01

Values are presented as the mean and standard deviation; 3-RM = three-repetition maximum test; N = Newton; J = Joule; SM = Smith machine; LP = leg press, HR = heart rate; RRsys = systolic blood pressure.

### Cardiovascular outcomes at baseline, during exercise, and postexercise periods in SM and LP

Table 2 illustrates the mean differences in cardiovascular metrics between the SM and LP conditions across various measurement time points (rest, exercise, immediate postexercise, and five minutes postexercise). The mean values for the SM condition demonstrated significant differences in cardiovascular variables at baseline compared with those of the LP condition (refer to Table 2). HR was notably greater during the SM condition when resting. In contrast, the SV, EDV, cardiac workload (CW), and TPR were significantly lower in the SM condition than in the LP condition. However, the SBP, DBP, cardiac output (CO), and EF did not significantly differ between the SM and LP conditions at rest. Significantly higher values of HR, CO, cardiac work (CW), SBP, and DBP were observed during exercise in the SM condition. The LP procedure resulted in significantly greater values of EDV and SV.

During the first postexercise minute, we found no differences in SBP, DBP, SV, or CW between conditions. However, the HR, CO, and EF were significantly greater under the SM condition, whereas the TPR was significantly lower than those under the LP condition.

**Fig. 2:**
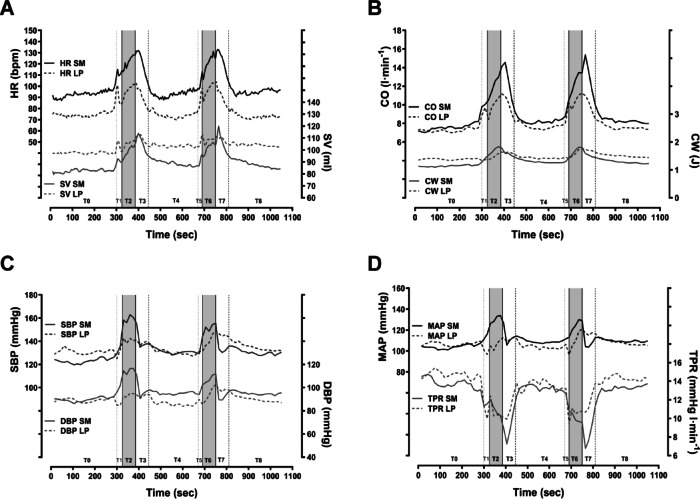
Graphs showing the mean cardiac response to squats in the Smith machine and leg bends in Leg Press throughout the training sessions (*n* = 14). (**A**) Heart rate and stroke volume; (**B**) cardiac output and cardiac work; (**C**) systolic blood pressure and diastolic blood pressure; D) mean atrial pressure and total periphery resistance.

**Fig. 3:**
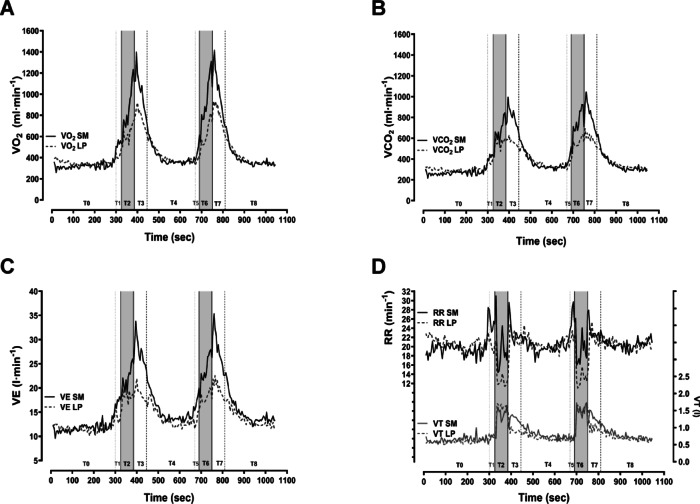
Graphs showing the mean pulmonary response to squats in the Smith machine and leg bends in Leg Press during the entire training session (*n* = 14). (**A**) Oxygen uptake; (**B**) carbon dioxide output; (**C**) ventilation; (**D**) respiratory rate and tidal volume.

At five minutes postexercise, there were no differences in SBP, DBP, EF, or CW between conditions. Nevertheless, the HR and CO values remained significantly greater under the SM condition, whereas the SV, EDV, and TPR were significantly lower under the SM condition than under the LP condition. Figures 2 and 3 illustrate the primary (cardiopulmonary and vascular response) and secondary (pulmonary response) outcomes during the examination period.

**Table 2:** Cardiovascular responses at rest, during exercise, and postexercise (two-way ANOVA with post hoc comparison test; *n* = 14; excluding the isometric period).

Cardiac and vascular outcomes	SM	LP	p value/post hoc-test	Effect size group effect	Main effects (group, time, interaction)		
	50% 3-RM	50% 3-RM					
				η^2^p	Group	Time	Interaction
					F value; p value	F value; p value	F value; p value
**SBP (mmHg)**				< 0.01			
Baseline	126 ± 11	132 ± 11	0.28				
Exercise	**157 ± 15**	**141 ± 10**	**< 0.01**				
1 min postexercise	137 ± 17	143 ± 9	0.14		F = 0.09;	**F = 25.9, **	**F = 11.8;**
5 min postexercise	134 ± 13.3	134 ± 7	> 0.99		p =.77	**p <.01**	**p <.01**
**DBP** **(mmHg)**				0.25			
Baseline	90 ± 9	90 ± 9	> 0.99				
Exercise	**111 ± 11**	**96 ± 8**	**< 0.01**				
1 min postexercise	91 ± 11	96 ± 11	0.16		F = 4.4;	**F = 16.7;**	**F = 15.1;**
5 min postexercise	93 ± 8	89 ± 7	0.54		p =.06	**p.<01**	**p <.01**
**MAP** **(mmHg)**				0.15			
Baseline	105 ± 9	108 ± 9	> 0.99				
Exercise	**129 ± 15**	**111 ± 12**	**< 0.01**				
1 min postexercise	108 ± 12	115 ± 8	0.09		F = 2.3;	**F = 16.0;**	**F = 17.1;**
5 min postexercise	109 ± 9	108 ± 10	> 0.99		p =.15	**p.<01**	**p <.01**
**HR** **(bpm)**				0.64			
Baseline	**91.5 ± 7.0**	**76.2 ± 9.4**	**< 0.01**				
Exercise	**123 ± 11.1**	**97.1 ± 7.1**	**< 0.01**				
1 min postexercise	**121 ± 12.4**	**91.7 ± 6.5**	**< 0.01**		**F = 162;**	**F = 139;**	**F = 13.3;**
5 min postexercise	**100 ± 10.2**	**78.7 ± 5.6**	**< 0.01**		**p <.01**	**p <.01**	**p <.01**
**SV** **(ml)**				0.18			
Rest	**83.4 ± 11.3**	**97.6 ± 8.8**	**< 0.01**				
Exercise	**102.3 ± 14.6**	**107 ± 7.3**	**< 0.01**				
1 min postexercise	108.4 ± 15.5	109 ± 7.5	> 0.99		F = 2.8;	**F = 41.1;**	**F = 18.6;**
5 min postexercise	**94.9 ± 14.0**	**105 ± 6.6**	**< 0.01**		p =.12	**p <.01**	**p <.01**
**CO** (l⋅min ^−1^)				0.56			
Baseline	7.5 ± 0.6	7.3 ± 1.0	> 0.99				
Exercise	**12.3 ± 1.9**	**10.3 ± 1.0**	**< 0.01**				
1 min postexercise	**13.0 ± 1.8**	**10.0 ± 1.0**	**< 0.01**		**F = 16.2;**	**F = 125;**	**F = 29.4;**
5 min postexercise	**9.4 ± 1.0**	**8.2 ± 0.8**	**< 0.01**		**p <.01**	**p <.01**	**p <.01**
**EDV ** **(ml)**				0.19			
Baseline	**118 ± 20.3**	**138 ± 15.3**	**< 0.01**				
Exercise	**134 ± 21.8**	**142 ± 14.9**	**< 0.01**				
1 min postexercise	**139 ± 22.9**	**146 ± 15.2**	**< 0.01**		F = 3.1;	**F = 41.3;**	**F = 19.2;**
5 min postexercise	**129 ± 23.8**	**143 ± 13.4**	**< 0.01**		p =.10	**p <.01**	**p <.01**
**CW** **(J)**				0.05			
Baseline	**1.2 ± 0.2**	**1.4 ± 0.1**	**< 0.01**				
Exercise	**1.8 ± 0.3**	**1.6 ± 0.1**	**< 0.01**				
1 min postexercise	1.6 ± 0.4	1.7 ± 0.2	0.32		F = 0.7;	**F = 47.5;**	**F = 12.8;**
5 min postexercise	1.4 ± 0.3	1.5 ± 0.1	0.1		p =.41	**p <.01**	**p <.01**
**TPR** (mmHg⋅ l⋅min ^−1^)				0.43			
Baseline	**14.0 ± 1.8**	**15.0 ± 2.7**	** 0.01**				
Exercise	10.7 ± 1.7	10.9 ± 1.5	> 0.99				
1 min postexercise	**8.5 ± 1.5**	**11.6 ± 1.6**	**< 0.01**		**F = 9.7;**	**F = 58.6,**	**F = 17.0**;
5 min postexercise	**11.8 ± 1.5**	**13.3 ± 1.8**	**< 0.01**		**p <.01**	**p <.01**	**p <.01**
**EF** **(%)**				0.03			
Baseline	71 ± 8	71 ± 6	> 0.99				
Exercise	77 ± 8	76 ± 7	0.93				
1 min postexercise	**78 ± 8**	**75 ± 7**	**< 0.01**		F = 0.4;	**F = 49.3; **	**F = 6.3;**
5 min postexercise	74 ± 8	74 ± 7	> 0.99		p =.54	**p <.01**	**p <.01**

Values are presented as the mean and standard deviation; F = effect size according to Cohen’s two-way repeated-measures ANOVA, η^2^p = partial eta square, SBP = systolic blood pressure; DBP = diastolic blood pressure; HR = heart rate; SV = stroke volume; CO = cardiac output; CW = cardiac work; EDV = end-diastolic volume, EF % = ejection fraction; TPR = total periphery resistance. The differences between the Smith machine (SM) and leg press (LP) methods are highlighted in bold (p <.05).

### Pulomary outcomes of SM and LP at baseline, during exercise, and during the postexercise period

Table 3 presents the mean pulmonary responses measured at baseline, immediately after exercise, and 5 min postexercise for both the SM and LP conditions. During the baseline period, no differences in pulmonary function were observed (as shown in Table 3). However, significant differences were detected during the exercise period, with notable increases in V_E_, respiratory rate (RR), VO_2_, VCO_2_, and RPE while the participants exercised in the SM condition. Additionally, there was a significantly stronger ventilatory response, as indicated by VO_2_, VCO_2_, V_E_, and tidal volume (VT), immediately after exercise in the SM condition. During the 5-minute postexercise period, V_E_ and VCO_2_ were significantly greater under the SM condition than under the LP condition.

**Table 3:** Pulmonary responses and RPE during the rest, exercise, and postexercise periods (two-way ANOVA with post hoc comparison test; *n* = 14; excluding the isometric period).

Pulmonary outcomes	SM	LP	p value/post hoc-test	Effect size group effect	Main effects (group, time, interaction)		
	50% 3-RM	50% 3-RM					
				η^2^p	Group	Time	Interaction
					F value; p value	F value; p value	F value; p value
**V** (l⋅ min^−1^)				0.87			
Baseline	**12.0 ± 1.9**	**11.7 ± 1.7**	**> 0.99**				
Exercise	**22.9 ± 2.8**	**18.0 ± 2.5**	**< 0.01**				
1 min postexercise	**27.5 ± 3.9**	**19.2 ± 2.2**	**< 0.01**		**F = 88.4;**	**F = 182**	**F = 48.4;**
5 min postexercise	**17.6 ± 1.9**	**14.7 ± 1.6**	**< 0.01**		**p <.01**	**p <.01**	**p <.01**
**RR** (breaths⋅ min ^−1^)				0.42			
Baseline	19.8 ± 3.0	20.8 ± 3.3	0.63				
Exercise	**19.5 ± 5.2**	**13.8 ± 3.1**	**< 0.01**				
1 min postexercise	23.1 ± 5.1	21.6 ± 3.3	0.15		F = 9.6;	F = 12.9;	F = 18.4;
5 min postexercise	21.0 ± 4.0	20.8 ± 3.5	> 0.99		p <.01	p <.01	p <.01
**VT** **(l)**				. 33			
Baseline	0.7 ± 0.1	0.6 ± 0.1	> 0.99				
Exercise	1.4 ± 0.4	1.5 ± 0.4	0.42				
1 min postexercise	**1.3 ± 0.3**	**1.0 ± 0.2**	**< 0.01**		**F = 6.5;**	**F = 44.4;**	**F = 10.1;**
5 min postexercise	0.9 ± 0.2	0.8 ± 0.1	0.06		**p =.02**	**p <.01**	**p <.01**
**VO**_2_ (ml⋅ min^−1^)				0.9			
Baseline	330 ± 39.2	350 ± 39.2	> 0.99				
Exercise	**901 ± 104.4**	**623 ± 65.1**	**< 0.01**				
1 min postexercise	**1005 ± 116**	**777 ± 66.2**	**< 0.01**		**F = 119.4;**	**F = 466;**	**F = 62.0,**
5 min postexercise	520 ± 43.8	478 ± 35.9	0.1		**p <.01**	**p <.01**	**p <.01**
**VCO**_2_ (ml⋅ min^−1^)				0.88			
Baseline	278 ± 41.9	298 ± 40.9	0.57				
Exercise	**705 ± 87.0**	**546 ± 79.3**	**< 0.01**				
1 min postexercise	**815 ± 0.97.1**	**575 ± 64.2**	**< 0.01**		**F = 90.1;**	**F = 252;**	**F = 70.4;**
5 min postexercise	**455 ± 38.9**	**407 ± 43.5**	**0.01**		**p <.01**	**p <.01**	**p <.01**
				0.06			
**RPE** **(1–10)**							
Baseline	1.0 ± 0.0	1.2 ± 0.4	> 0.99				
Exercise	**4.5 ± 1.3**	**3.6 ± 1.0**	**0.01**		F = 0.89;	**F = 153.3;**	**F = 4.7;**
3 min postexercise	1.3 ± 0.5	1.4 ± 0.5	> 0.99		p =.37	**p <.01**	**p =.02**

Values are presented as the mean and standard deviation, F = Effect size according to Cohen’s two-way repeated-measures ANOVA, η^2^p = partial eta square, V_E_ = ventilation; RR = respiratory rate, VT = tidal volume, VO_2_ = oxygen uptake; VCO_2_ = carbon dioxide output; RPE = rating of perceived exertion. Printed boldly (p <.05) different from the Smith machine (SM) vs. the leg press (LP).

### Changes in primary and secondary outcomes in each period in relation to the previous period

Table 4 illustrates the differences between the mean values of the exercise period, 1 min postexercise, and 5 min postexercise in relation to the rest of the period.

**Fig. 4:**
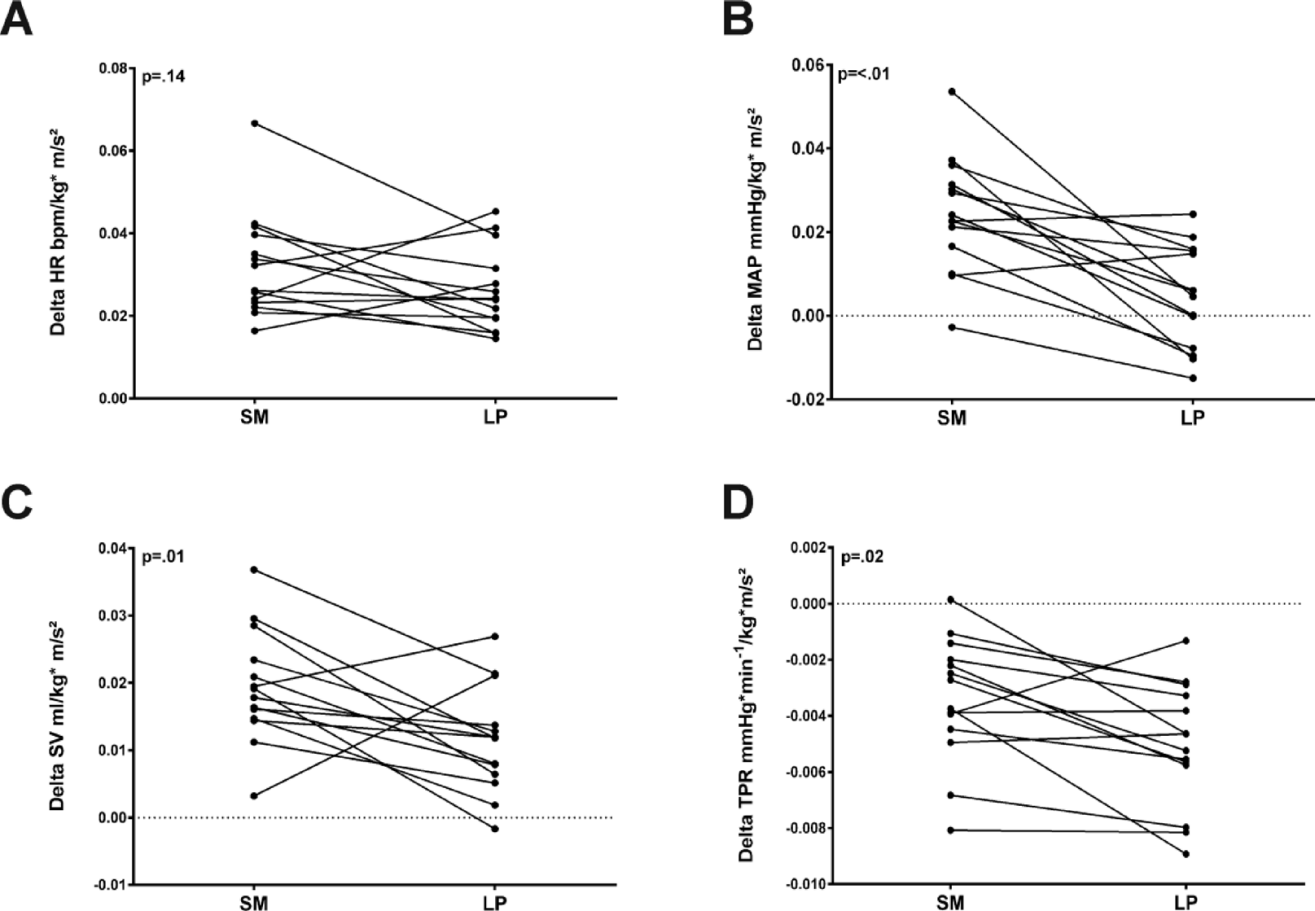
Graphs showing the changes for (**A**) HR; (**B**) MAP; (**C**) SV; and (**D**) TPR from rest to exercise in relation to the mean ground reaction force during squats in the Smith machine and leg bends in the Leg Press test (*n* = 14).

Figure 4 illustrates how the outcome changes in relation to the mean ground reaction force. The increase in HR was not significantly associated with the ground reaction force. In contrast, TPR, SV, EDV, and MAP demonstrate significantly greater increases that are correlated with the measured ground force.

**Table 4:** Mean change in cardiac and vascular outcomes compared with the previous measurement timepoint (∆ values = current periods minus previous period; *n* = 14).

∆cardiac and vascular outcomes	SM 50% 3-RM	LP 50% 3-RM	p value/post hoc-test	Effect size	Main effects (group, time, interaction)		
				η^2^p	Group	Time	Interaction
					F value; p value	F value; p value	F value; p value
**HR (bpm)**				0.64			
∆ Exercise	**31.5 ± 11.6**	**20.9 ± 6.9**	**< 0.01**				
∆1 min postexercise	−2.4 ± 8.8	−5.4 ± 3.5	0.97		**F = 23.0;**	**F = 144.7;**	**F = 9.3;**
∆5 min postexercise	−20.4 ± 4.6	−13.0 ± 4.5	0.06		**p <.01**	**p <.01**	**p =.01**
**SV (ml)**				0.29			
∆ Exercise	**18.9 ± 7.6**	**9.2 ± 6.2**	**< 0.01**				
∆1 min postexercise	6.1 ± 7.8	2.0 ± 5.2	0.31		**F = 5.2;**	**F = 70.5;**	**F = 17.6;**
∆5 min postexercise	**−13.5 ± 3.9**	**−3.9 ± 3.5**	**< 0.01**		**p =.04**	**p <.01**	**p <.01**
**EDV (ml)**				0.37			
∆ Exercise	**16.2 ± 7.3**	**4.1 ± 6.2**	**< 0.01**				
∆1 min postexercise	5.1 ± 7.3	3.6 ± 5.7	> 0.99		**F = 7.6;**	**F = 38.3;**	**F = 9.0;**
∆5 min postexercise	**−10.5 ± 4.0**	**−2.4 ± 4.3**	**< 0.01**		**p =.02**	**p <.01**	**p <.01**
**CW (J)**				0.09			
∆ Exercise	**0.6 ± 0.3**	**0.2 ± 0.2**	**< 0.01**				
∆1 min postexercise	**−0.2 ± 0.15**	**0.08 ± 0.16**	**< 0.01**		F = 3.6;	**F = 58.8;**	**F = 21.1;**
∆5 min postexercise	−0.2 ± 0.10	−0.2 ± 0.13	> 0.99		p =.08	**p <.01**	**p <.1**
**MAP (mmHg)**				0.12			
∆ Exercise	**24.2 ± 14.5**	**3.3 ± 9.5**	**< 0.01**				
∆1 min postexercise	**−20.9 ± 9.9**	**3.3 ± 11.0**	**< 0.01**		F = 1.8;	**F = 21.5;**	**F = 36.3;**
∆5 min postexercise	0.8 ± 6.6	−7.0 ± 7.6	0.16		p =.21	**p <.01**	**p <.01**
**TPR** (mmHg⋅ l⋅min^−1^)				0.09			
∆ Exercise	**−3.3 ± 2.1**	**−4.1 ± 1.8**	**0.16**				
∆1 min postexercise	**−2.3 ± 1.7**	**0.7 ± 1.3**	**< 0.01**		**F = 1.2;**	**F = 55.3;**	**F = 37.4;**
∆5 min postexercise	**3.3 ± 0.8**	**1.7 ± 0.8**	**< 0.01**		**p =.29**	**p <.01**	**p <.01**

Values are presented as the mean and standard deviation; F = Effect size according to Cohen’s two-way repeated-measures ANOVA; η^2^p = partial eta square; ∆= average changes in the mean values of the outcomes between the periods; printed boldly (p <.05) different from the Smith machine (SM) vs. the leg press (LP).

## Discussion

The main findings of this randomized crossover study are as follows:

I) At rest, HR and SV significantly differed between SM and LP while the same CO was maintained.
II) During exercise, squats in the SM produce greater ground reaction forces (muscular load). They elicited a more pronounced orthostasis- and intensity-dependent cardiopulmonary response (including increased blood pressure, HR, CO, and VO_2_) at a TPR similar to that of the LP.
III) SM facilitates a significant intensity-related reduction in the TPR, thereby helping maintain blood pressure despite having higher CO than does LP during the postexercise period.

### Baseline regulation (rest period)

Our data revealed significant HR, SV, and TPR differences when the standing SM was compared with the LP. The pulmonary values remain similar. Blood pressure regulation and orthostatic regulation are clearly understood when adjusting positions that have a significant impact from gravity^36^. Rowell (1993) suggested that transitioning to an upright position causes a shift in central blood volume to peripheral areas, leading to a decrease in both SV and CO^36^. The baroreflex plays a crucial role in compensating for decreased blood volume or reduced venous return when standing. When blood pressure decreases, the body responds with the baroreflex, which increases the HR. This happens due to decreased parasympathetic activity and increased sympathetic vasoconstrictor activity, resulting in a higher TPR^22,36,37^. After these adjustments, the blood volume remains low, whereas the TPR increases. This elevation affects primary DBP and is linked to decreased SV^36,37^. Schwartz and Stewart (2012) demonstrated that changing position has a minor effect on CO, unlike the significant reduction observed in SV^37^. Adjusting the stimulus‒response curve is crucial for regulating the baroreflex response to orthostatic and exercise-induced stress, thereby preventing postural hypotension^37,38^. In contrast to the findings of some studies, we found that the TPR was significantly greater under LP conditions than under SM conditions. Baum et al. (2003) found that raising the feet during a LP exercise increased blood pressure and proposed that a short-term isometric contraction contributed to this effect. This phenomenon is probably related to the seated position with the horizontal positioning of the legs during the LP execution^39,40^. Similarly, no evidence exists of a change in the baroreflex response at rest on ventilation outcomes^37^. In summary, cardiovascular differences in HR and SV occur on the basis of body position at rest^36,37^.

### Cardiac and vascular regulation during exercise

With matched weight loads (50%−3RM), the lower work rate efficiency shown for the SM confirms the previous assumption that the standing exercise in the SM requires greater muscle effort and likely greater stability demands on the trunk muscles than the LP exercise does^18,41^.

We observed significantly greater SBP and DBP in the SM group than in the LP group (Table 3). A systematic review by Blazek et al. (2019) indicated that intra-abdominal pressure is substantially greater during standing squats than during LPs. On the basis of these findings, the authors recommend using the LP over the SM^20^. Since these studies also used the Valsalva manoeuvre and did not directly compare LPs with SMs, the results cannot be directly compared with those of the present study. We systematically assessed the frequency, volume, and pressure regulation to better understand the differences in blood pressure.

*Heart rate*: During exercise, the increase in ∆HR was significantly greater in the SM than in the LP (see Table 1). Figure 4 shows that the relationship between the ∆HR and ground reaction force (∆HR/GRF) did not differ significantly between the SM and LP conditions. A key finding is that the greater HR increase in SM is related to body position rather than exercise intensity (GRF). Zhang et al. (2009) reported that blood pressure and HR fluctuations during position changes in a single squat-stand manoeuvre are mediated by the baroreflex mechanism, with no significant differences in baroreflex sensitivity across exercise intensities^42^. Some studies have shown that the arterial baroreflex is reset during static and dynamic strength exercises. Furthermore, both the feedforward mechanism of central control and the feedback mechanism associated with the afferents of the skeletal muscles (the exercise pressure reflex) play a central role in this exercise adaptation^43,44^. When standing, greater isometric muscle work (lower work efficiency) could trigger a greater pressure reflex response during exercise and thus may explain the increased cardiac responses^45,46^. The elevated resting HRs and greater increase in HR during squatting are due primarily to the upright position and the greater sympathetic nerve activation (SNA) caused by body position depended intensity. In line with the study by Sousa et al. (2014), our data (Fig. 2) indicate that both blood pressure and HR decrease at the start of LP^40^. This initial drop in HR is likely due to baroreflex-mediated short-term responses, as we observed a significantly greater SV during LP.

*Stroke volume*: During exercise, we observed that the changes in the SV and EDV were significantly greater in the SM than in the LP. When considering the GRFs and the differences in intensity between SM and LP (see Fig. 4), the increases in SV and EDV (∆EDV/GRF; ∆SV/GRF) were greater in SM. This greater increase in SV appears to be due primarily to the greater intensity experienced during the SM. Katayama et al. (2018) reported that muscle sympathetic nervous system (MSNA) activation increases with exercise intensity, whereas the metaboreflex reduces the ability of the cardiopulmonary baroreflex to inhibit sympathetic activity. They also noted that low-intensity dynamic exercise, which increases central blood volume, suppresses MSNA and significantly lowers the rise in exercise-induced blood pressure^47^. This may explain the lower blood pressure values observed during LP conditions, as the EDV is significantly greater while the net intensity is lower. Additionally, the absolute mean values for SV and EDV are notably lower during both exercise and resting conditions when squats are performed in an SM^36,37,48^, which is attributable to body position.

*Total peripheral resistance*: During exercise, the decrease in ∆TPR was not significantly different between the LP and SM conditions. However, we observed a significantly smaller reduction in the SM condition when the ground reaction force (∆TPR/GRF) was considered. The absolute values of TPR did not differ between SM and LP during loading. An increased feedback exercise pressure reflex response to muscle sympathetic nerve activity (MSNA) could explain the smaller decrease in TPR observed with SM, which may be triggered by higher intensity during SM^47,49^. Sousa et al. (2014) reported that blood pressure increases linearly while squatting with 50% of the one-repetition maximum (1RM) in the LP after an initial drop, peaking at the end of exercise. This pattern, influenced by exercise intensity, is linked to rising CO and TPR. This is supported by the findings of the present study, which show that blood pressure initially decreases during the LP before rising only moderately during the first set (see Fig. 2)^40^. During the second set, blood pressure increases more significantly after an initial drop. This aligns with the observed increase in blood pressure during repetitive stress exercises (isotonic variants) in weightlifting, which may initially lower the TPR and explain the concomitant reduction in blood pressure. However, the authors propose that the rise in blood pressure during exercise is primarily linked to increased TPR, similar to what occurs during isometric strength exercises^39,40^. Our data indicate a decreasing TPR during exercise for both LPs and SMs (see Fig. 2). Consequently, the simultaneous increase in blood pressure appears to be driven by CO. The squats performed in the SM lead to higher CO values, which can explain the elevated SBP.

*Pulmonary exercise regulation*: During exercise, the VO_2_, VCO_2_, and V_E_ levels in SM were significantly greater. These results indicate greater metabolic demands for SM, leading to stronger ventilatory responses^46^. Interestingly, V_E_ during exercise is mediated by a higher RR in SM. A stronger baroreflex reset does not appear to stimulate the ventilatory increase by tidal volume, as previously hypothesized^37^. Since the V_E_ values were identical in the baseline situation, the differences in V_E_ during exercise appear to be influenced by the greater intensity and likely greater resetting of the baroreflex in SM. This effect may be mediated by exercise pressure reflex responses, involving afferent sensory neurons in both the group IV (metabolic) and group III (mechanical) pathways^43,46,49^. However, both energy expenditure and VO_2_ are known to increase depending on the muscle mass used^50^. Thus, the increased gas exchange parameters and ventilation parameters during exercise are consistent with the decreased work efficiency rate and the increased oxygen demands for SM performance.

*In summary*, there were significant differences in cardiac outcomes (HR, SV, CO, SBP, and DBP) between SM and LP, whereas there were no differences in TPR. The notably higher CO in SM is partly triggered by body position-dependent frequency adaptation^42^. However, the greater increase in SV during exercise in the SM is primarily due to the greater intensity. This also results in a more pronounced ventilatory response and suggests that a stronger baroreflex is reset in the SM^43,45,49^. The higher blood pressure in the SM results from greater activation of the SNA, resulting in vigorous peripheral afferent feedback related to intensity^42,45,49^.

### Immediate postexercise regulation

Immediately after the exercise period, we observed no differences in blood pressure. However, there were clear differences in HR, CO, and TPR between SM and LP. Compared with that under LP, the absolute HR under SM was significantly greater. The ∆HR from exercise to the postexercise condition was similar for both conditions. Consistent with the current literature, our data indicate that the greater intensity and greater orthostatic stress associated with squats in the SM lead to a stronger HR-mediated response (enhanced baroreflex reset), resulting in increased CO during the immediate postexercise period^45,49,51^. During the transition from exercise to rest, the change in SV was similar for both the LP and SM groups. The significantly higher CO observed in the SM group is primarily due to a higher absolute HR. According to Rezek et al. (2006), the decrease in blood pressure following exercise is attributed mainly to the decline in CO, whereas the TPR remains unchanged^52^. The present data indicate a clear increase or stable CO for SM and LP, respectively.

*The TPR and the change in TPR* (∆TPR) were significantly different between the LP and SM conditions immediately after exercise. Under the SM condition, the TPR decreased significantly by 2.3 mmHg⋅l⋅min, whereas it increased by 0.7 mmHg⋅l⋅min under the LP condition. The current literature indicates that isometric strength training is most effective at reducing resting blood pressure, which is linked to a lower TPR^12,13,26^. Both isometric training and, to a lesser extent, dynamic strength training appear to reduce postexercise blood pressure^26^. Our data indicate that despite having a significantly greater HR in the SM condition and the same SV, the TPR in SM decreased significantly more during the immediate postexercise period. As a result of this change in the TPR, the mean arterial pressure (MAP) also significantly decreased in the SM condition during the first minute after exercise, with a change in the MAP (∆MAP) of −20.9 ± 9.9 mmHg. In contrast, the MAP increased under the LP condition, with a change of + 3.3 ± 11.0 mmHg. This reduction in afterload under the SM condition is notable, even though CO was significantly greater than that under the LP condition. Edwards et al. (2022) suggested that increased autonomic vasomotor activity during squat exercise enhances the reduction in the TPR^26^. However, our data suggest a higher HR level (more active SNA) in the standing position. Thus, the reduction in TPR in the standing position seems to be the result of the higher metabolic demands shown, which explains the reduction in postexercise blood pressure behavior^53–55^. Furthermore, given that no difference in SBP or DBP was observed between conditions during the first minute after exercise, these regulatory differences may be interpreted as acute stabilization of blood pressure in response to stress.

*Ventilatory outcomes* were significantly greater in SM than in LP immediately after exercise. These findings suggest that vasomotor activity is stronger in SM. The increased activation of the peripheral muscle metaboreflex in the SM may help reset the baroreflex^49^, which also helps to explain the significantly greater respiratory response^46^. In the first minute following exercise, the increased V_E_ in the SM group can be attributed to a significantly greater tidal volume (VT). However, the extent to which this metabolically induced stimulation affects the ventilatory baroreflex response—and consequently supports ventilation and CO—remains largely hypothetical^37,56^.

*In summary*, the absolute values observed during the immediate postexercise period show a similar pattern to those observed during the exercise period. Notably, there is a distinct change in the TPR and MAP between the SM and the LP conditions. In line with the hypothesis proposed by Kamiya et al. (2005), the resetting of the baroreflex due to orthostatic stress, along with a more pronounced metaboreflex in the SM, may compensate for the lower TPR observed immediately after exercise, thereby preventing postural hypertension^38^. Assuming that intensity reflects muscle contraction, greater intensity increases acute MAP and causes a more pronounced postural drop in blood pressure^57^. This confirms the assumption and demonstrates that the anticipated cardiovascular requirements are more significant under SM, leading to notably higher afterload responses than under LP.

### 5-Minute postexercise regulation

During the recovery period, which lasts up to 5 min after exercise, cardiopulmonary parameters return to resting levels. While SBP and DBP were comparable between conditions, HR was greater in the SM. This difference may be attributed to differences in body position. Compared with that in the resting period, CO in the SM period was significantly greater than that in the LP period because of increased load deflection. Although TPR (denoted as ∆TPR) increased significantly more for SM than for LP, SM presented notably lower absolute TPR values during the 5-minute postexercise period.

Additionally, under SM conditions, significantly greater changes in ∆SV and EDV were observed. In contrast, the change in ∆HR was not significantly different between the two conditions during the recovery period. The increased CO during the postexercise period in SM results from a higher absolute HR, with blood pressure being maintained by a significantly lower TPR.

In summary, cardiopulmonary outcomes clearly show orthostatic regulation during the gradual recovery phase; however, the increased training load during SM is clearly reflected in the postexercise response.

### Derivation for health sports

Compared with subjectively matched LP training, exercising in an SM revealed greater muscular and cardiopulmonary demands and may also result in stronger long-term adaptations^15,18,50^. The increased HR at baseline in SM is orthostatic-dependent and is maintained because of the greater physical effort during the exercise period. In conclusion, chronic functional and morphological adaptations of the heart may occur in both healthy and diseased individuals^1,8,11,58^. During LP, there is a consistently greater EDV and SV, which may also indicate acute mechanisms. These acute responses are typical of dynamic exercise, which does not significantly increase blood pressure and can result in functional or eccentric hypertrophy of the myocardium^9^. However, Lovic et al. (2017) suggested that a SBP greater than 150 mmHg or 60% of peak exercise capacity serves as the threshold for cardiopulmonary remodelling. Therefore, LP exercises may also lead to improvements in individuals or patients with reduced cardiopulmonary capacity.

In addition, there was a significant difference in the exercise blood pressure response between the SM and LP groups. Despite no change in the decrease of TPR, the greater increase in CO contributes to the larger rise in blood pressure observed in the SM condition, which is crucial for high-risk patients to consider^20,59^. However, the elevated exercise blood pressure in SM also caused more pronounced postexercise hypotension^13^. In summary, data from healthy participants indicate that cardiovascular and pulmonary responses are more pronounced in SM squatting than in subjectively intensity-matched exercise in the LP. Conversely, the risk of blood pressure peaks is much lower with LP. The decision between the two exercise types (SM vs. LP) should be tailored to each patient on the basis of their specific indications and risk factors.

### Study limitations

Several limitations should be recognized, as they may impact the interpretation of the study findings. First, the sample size was relatively small and focused exclusively on active female participants to minimize any potential interference from sex differences in cardiopulmonary function and muscle performance. As a result, the interpretability and generalizability of the findings are limited and cannot apply to older or ill individuals. Therefore, conducting future studies with diverse patient groups is essential. Nevertheless, this trial is the largest randomized crossover study investigating acute cardiopulmonary responses during matched standing and sitting strength training exercises. Second, hormonal contraceptives and the menstrual cycle can influence blood pressure in females^24,60^. Since 35% of the participants did not report hormonal contraception, this could affect the results. Nevertheless, the study group was homogeneous and without physical complaints during the study visits. Third, while force plates are considered the “gold standard” for measuring ground reaction force, the measurement system used in the present study demonstrates high accuracy in measuring ground reaction force compared with force plates^61,62^. Fourth, ground reaction forces varied between the two exercises, affecting the muscular load. These differences in intensity may obscure the variations in body position related to cardiopulmonary regulation between the SM and LP. In discussing cardiovascular observations, autonomic regulatory mechanisms are particularly referenced to explain these findings. Fifth, using 3RM estimates instead of 1RM can lead to inaccurate assessments of training intensity; however, it was applied for safety reasons.

## Conclusions

Cardiopulmonary responses were found to be related to body position and exercise intensity. Squats performed in a SM result in greater cardiopulmonary effort and a stronger vascular response by lowering the TPR during the postexercise period. Compared with standing squats, exercising on a LP with similar subjective effort resulted in a greater stroke volume and significantly lower blood pressure. Future studies should evaluate how cardiopulmonary regulation differs between SM and LP exercises in cardiac patients.

## Data Availability

The datasets used and/or analysed during the current study available from the corresponding author on reasonable request.

## Abbreviations

CO: Cardiac output
CW: Cardiac work
DBP: Diastolic blood pressure
EDV: End diastolic volume
EF%: Ejection fraction
GRF: Ground reaction force
HR: Heart rate
LP: Leg press
MAP: Mean arterial pressure
3-RM: Three repetition maximum
RPE: Rating of perceived exertion
RPP: Rate pressure product
RR: Respiration rate
SBP: Systolic blood pressure
SM: Smith machine
SV: Stroke volume
TPR: Total peripheral resistance
V_E_: Minute ventilation
VCO_2_: Carbon dioxide output
VO_2_: Oxygen uptake
VT: Volume tidal
